# A novel fluorescein-bisphosphonate based diagnostic tool for the detection of hydroxyapatite in both cell and tissue models

**DOI:** 10.1038/s41598-018-35454-9

**Published:** 2018-11-26

**Authors:** Alisia M. Sim, Nabil A. Rashdan, Lin Cui, Alastair J. Moss, Fabio Nudelman, Marc R. Dweck, Vicky E. MacRae, Alison N. Hulme

**Affiliations:** 1 EaStCHEM School of Chemistry, University of Edinburgh, David Brewster Road, Edinburgh, EH9 3FJ UK; 2 The Roslin Institute, Royal (Dick) School of Veterinary Studies, University of Edinburgh, Easter Bush, Midlothian, EH25 9RG UK; 3 Centre for Cardiovascular Science, University of Edinburgh, Chancellor’s Building, Edinburgh, EH16 4UU UK

## Abstract

A rapid and efficient method for the detection of hydroxyapatite (HAP) has been developed which shows superiority to existing well-established methods. This fluorescein-bisphosphonate probe is highly selective for HAP over other calcium minerals and is capable of detecting lower levels of calcification in cellular models than either hydrochloric acid-based calcium leaching assays or the Alizarin S stain. The probe has been shown to be effective in both *in vitro* vascular calcification models and *in vitro* bone calcification models. Moreover we have demonstrated binding of this probe to vascular calcification in rat aorta and to areas of microcalcification, in human vascular tissue, beyond the resolution of computed tomography in human atherosclerotic plaques. Fluorescein-BP is therefore a highly sensitive and specific imaging probe for the detection of vascular calcification, with the potential to improve not only *ex vivo* assessments of HAP deposition but also the detection of vascular microcalcification in humans.

## Introduction

Calcium is a crucial intracellular element that is responsible for regulating many cellular processes across every cell type in biological organisms^1^. Calcium is found in either the free ion form or as a mineral phase, for example in the form of hydroxyapatite (HAP) that makes up bone and teeth. However mineralisation is also a critical component of a wide range of diseases such cancer, arthritis and cardiovascular disease (CVD)^2–4^. In atherosclerosis the presence of microscopic deposits of HAP can weaken the fibrous cap of an atherosclerotic plaque, leading to rupture of the plaque and vessel occlusion^5–9^. In aortic stenosis the progressive accumulation of HAP in the valve leads to increasing obstruction to the flow of blood out of the heart, whilst in abdominal aortic aneurysm disease microcalcification is associated with aneurysms that expand more quickly and are at increased risk of rupture or requiring repair. Thus the development of a calcium assay which is able to selectively detect HAP with high sensitivity and specificity could both improve understanding of disease pathophysiology and aid the diagnosis of a range of clinical disorders including vascular calcification.

**Figure 1:**
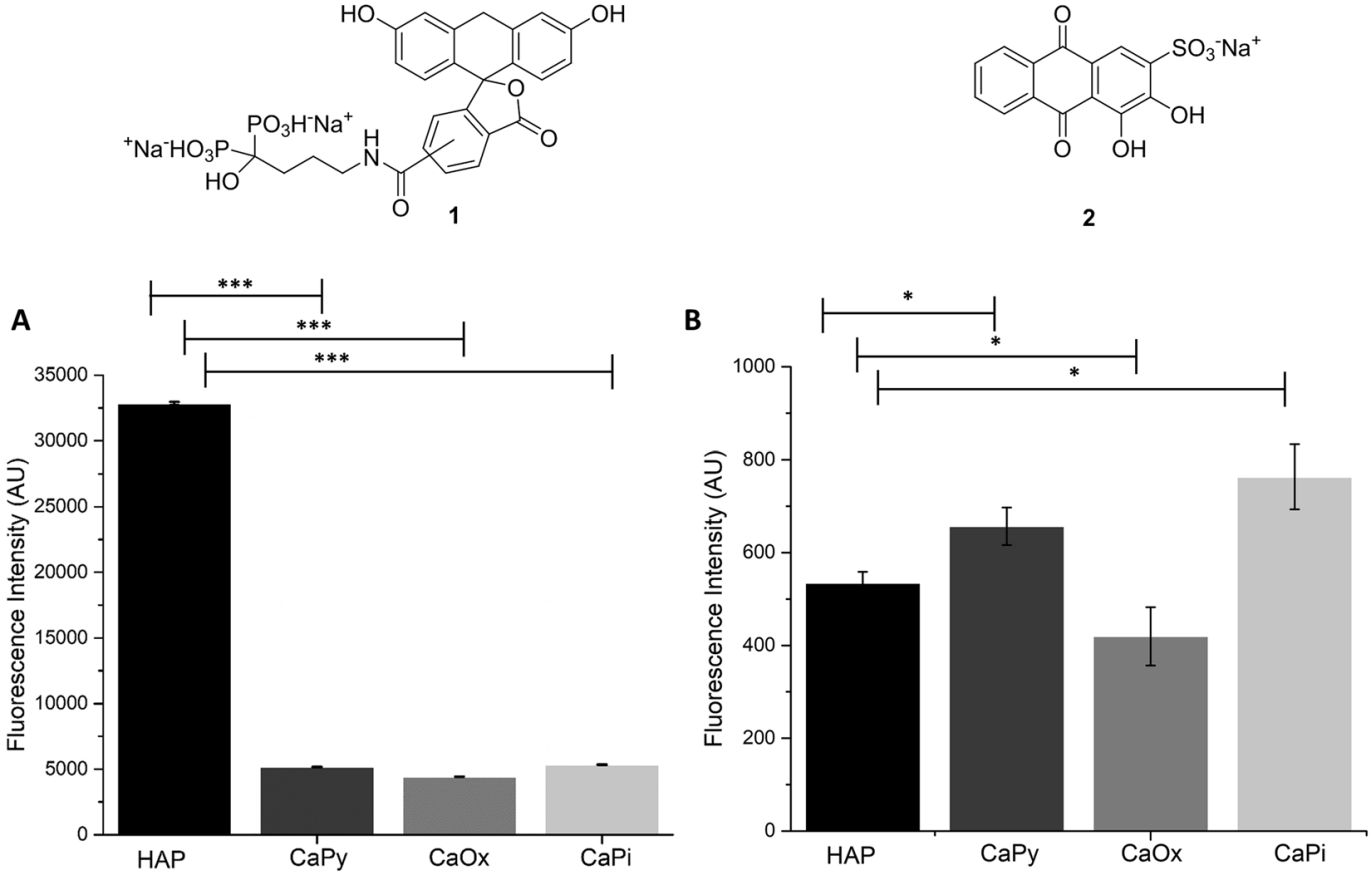
Selectivity towards calcium minerals of Fluorescein-BP (**1**) in comparison to Alizarin S stain (**2**). Calcium minerals were incubated with Fluorescein-BP (5 μM) (**A**) or 1% w/w Alizarin S (**B**) in water for 2 hours and the un-bound probe was removed and quantified. The histograms show the relative amounts of bound fluorophore for each of the calcium minerals. Data shown are representative of at least 3 independent experiments yielding comparable results. *P < 0.05, **P < 0.01, ***P < 0.001 compared to HAP, n = 6.

The most common assays for calcium phosphate mineralisation are calcium leaching by hydrochloric acid (HCl); and staining with Alizarin S and von Kossa stains^10,11^. Each comes with their own limitations. The calcium leaching assay is used in laboratory settings to determine the presence of calcification in vascular and bone cell models. The assay involves incubating cell monolayers in the presence of HCl which allows the extraction of free calcium ions from the cells. The calcium content is then detected using a colourimetric assay and normalised against the total number of cells per sample^12^. Given the timeframe of the calcium leaching assay (24–48 h) it is not suitable for high-throughput applications. The colourimetric stain Alizarin S detects calcium, and not phosphate, thus it can also bind to calcium-binding proteins and proteoglycans without discriminating for the presence of HAP^13^. Finally, the colourimetric von Kossa stain detects phosphates but only in acidic environments, even though the presence of phosphate does not necessarily imply the presence of calcium or even HAP^10,13^. Both Alizarin S and von Kossa stains are used to indicate calcium depositions but at best give only semi-quantitative calcium readings^14^. In this study, the fluorescein-bisphosphonate conjugate **1** (Fluorescein-BP, Fig. 1)^15^ was repurposed to allow for a rapid and inexpensive method of studying calcification and its use was investigated in a range of cell based models as well as mouse and human vascular tissue.

## Results

### Fluorescein-BP synthesis and specificity

The bisphosphonate, alendronate is recognised as a bone targeting reagent due to its striking ability to bind to HAP^16,17^. Previous experiments have shown that alendronate is readily able to bind to bulk calcium mineral even in the presence of free calcium ions; and it is approved for clinical use for Paget’s disease and osteoporosis^16,18,19^. The ready availability of the FDA-approved probe components makes the alendronate-based Fluorescein-BP conjugate **1** particularly attractive for development and human translation as a method for the detection of calcification. Recently the Leong group reported the use of Fluorescein-BP **1** for the study of urinary calculi^15^; although the fluorescence intensity of the probe in the presence of macroscopic calcium oxalate (CaOx) was found to be relatively moderate.

Fluorescein-BP was synthesised following the procedure reported by the Leong group^15^. Specificity of the probe towards HAP was shown by incubating Fluorescein-BP with a range of calcium minerals including HAP, calcium pyrophosphate (CaPy), CaOx and calcium phosphate (CaPi). Aqueous suspensions of these minerals at fixed weight per volume were incubated with probe **1** for 2 hours, and un-bound probe was removed from the solids through a series of centrifugation and washing steps. Fluorescence analysis of the combined aqueous extracts allowed the levels of un-bound probe to be quantified and hence a direct comparison of the levels of Fluorescein-BP binding to each of the different calcium-containing minerals was enabled. These experiments showed that Fluorescein-BP demonstrated almost an order of magnitude higher selectivity towards HAP than any other calcium species tested (Fig. 1A). In contrast, when Alizarin S (**2**, Fig. 1) the most common histological stain for calcium, was incubated with the different calcium minerals it showed a very similar fluorescent intensity for each indicating non-specific binding (Fig. 1B). In order to achieve effective visualisation of the Alizarin S stain by fluorescence, millimolar concentrations of the stain were required which is in sharp contrast to the low micromolar concentrations required of probe **1**. This reflects the relative brightness of the signal for each fluorophore which is proportional to its quantum yield (Φ = 0.79 for fluorescein^20–22^, and Φ = 0.001 for Alizarin S^22,23^).

### Use of Fluorescein-BP in cell-based models of vascular calcification

A series of experiments were designed to probe the ability of the Fluorescein-BP probe **1** to define the location of HAP in cellular calcification assays, exploiting both the selectivity and sensitivity which was demonstrated in the mineral-based assays. Vascular smooth muscle cells (VSMCs), the principal cell type involved in vascular calcification, can undergo a phenotypic transition under specific environmental conditions to calcifying osteoblastic cells^24^. The mouse VSMC line MOVAS-1 exhibits a smooth muscle cell phenotype and has been previously employed to investigate the VSMC cell cycle^25^. Validation of the Fluorescein-BP probe **1** was carried out using MOVAS-1 and primary mouse VSMCs.

**Figure 2:**
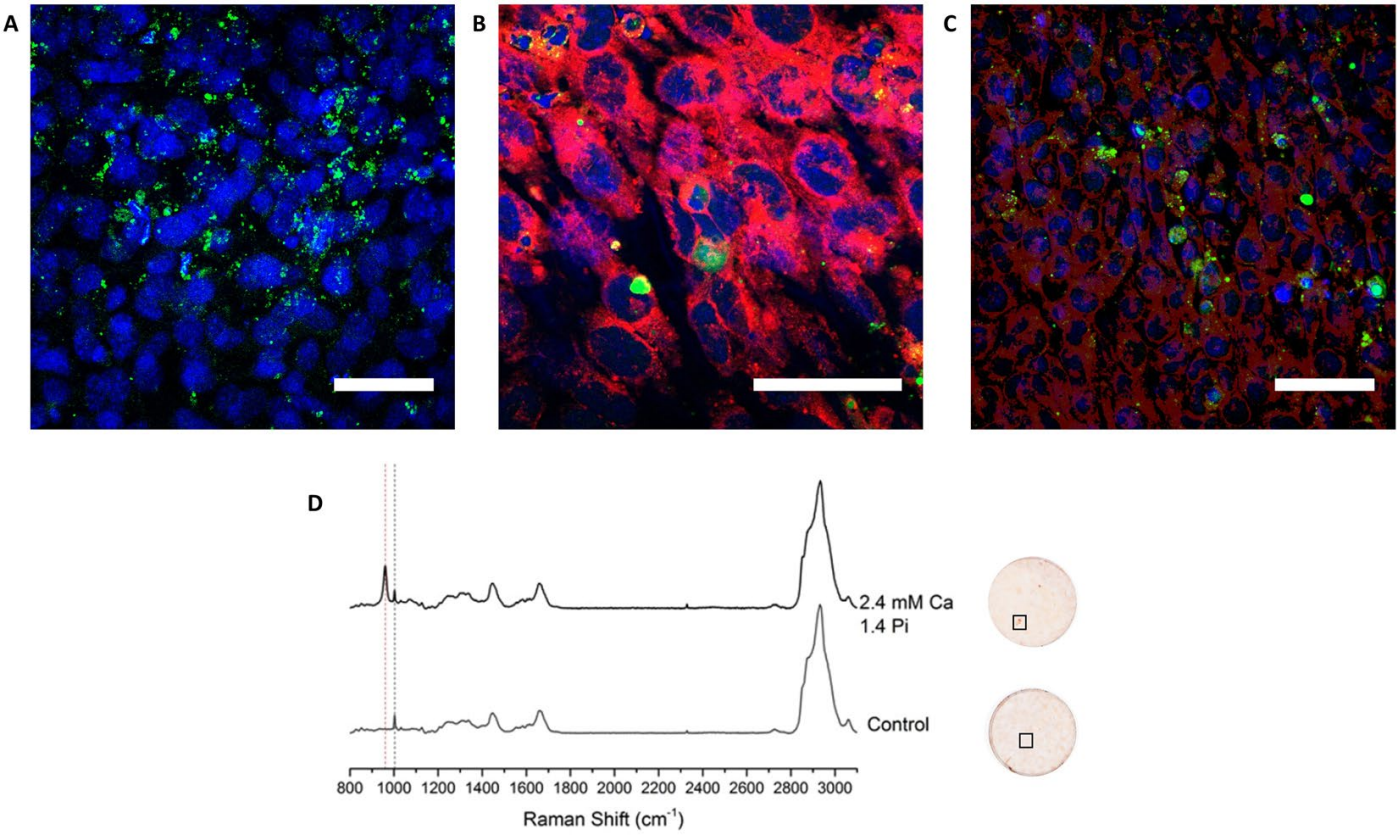
Confocal Images showing dual staining and Raman spectra of MOVAS-1 monolayers. Monolayers of MOVAS-1 cells were grown to confluence (day 0) and then switched to calcification media (2.4 mM Ca and 1.4 mM Pi) for 7 days, changing media on alternate days. The cell monolayers were treated sequentially with Fluorescein-BP (1 μM, 2 hours; green); CellMask Orange (500 nM, 10 minutes, pink); NBF (10%, 15 minutes); Triton X (0.05%, 3 × 5 minutes); DAPI (300 nM, 5 minutes, blue); then imaged on an Zeiss Confocal LSM710 microscope (λ_ex_ = 488 nm, 554 nm and 350 nm). Images are representative of at least 3 repeats. Scale bars = 100 µm. (**A**) Incubation with Fluorescein-BP and DAPI. (**B**,**C**) Incubation with Fluorescein-BP, CellMask Orange and DAPI. (**D**) Monolayers of MOVAS-1 cells were grown to confluence (day 0) on CaF_2_ slides and then switched to either control media or calcification media (2.4 mM Ca and 1.4 mM Pi) for 7 days, changing media on alternate days. The cell monolayers were fixed with NBF (10%, 15 minutes); and imaged using an InVia Renishaw Raman instrument (785 nm, 30 s, 50%). Following imaging, the cell monolayers were incubated with Alizarin S (2%, 10 minutes). Red dashed line indicates peak at 960 cm^−1^ for HAP while the black dashed line indicates the inherent cellular peak at 1003 cm^−1^ for phenylalanine. Images shown are representative of at least 3 independent experiments yielding comparable results.

Monolayers of MOVAS-1 cells were incubated with 2.4 mM Ca and 1.4 mM Pi for 7 days; then treated with Fluorescein-BP **1** for 2 h prior to imaging. Confocal microscopy (Fig. 2A–C) shows extracellular localisation of probe **1** (in green); indicative of the presence of HAP in the extracellular matrix in accord with previous reports^26–29^. In contrast, no signal was observed when the same cells were incubated with a fluorescein-amino-1-pentanol conjugate (SI Fig. 1); giving a clear indication that the bisphosphonate motif is responsible for Fluorescein-BP binding to HAP in a cellular environment. Raman spectroscopic analysis of the observed mineral deposits confirmed the presence of HAP, with its distinctive peak at 960 cm^−1^ (Fig. 2D)^30^.

**Figure 3:**
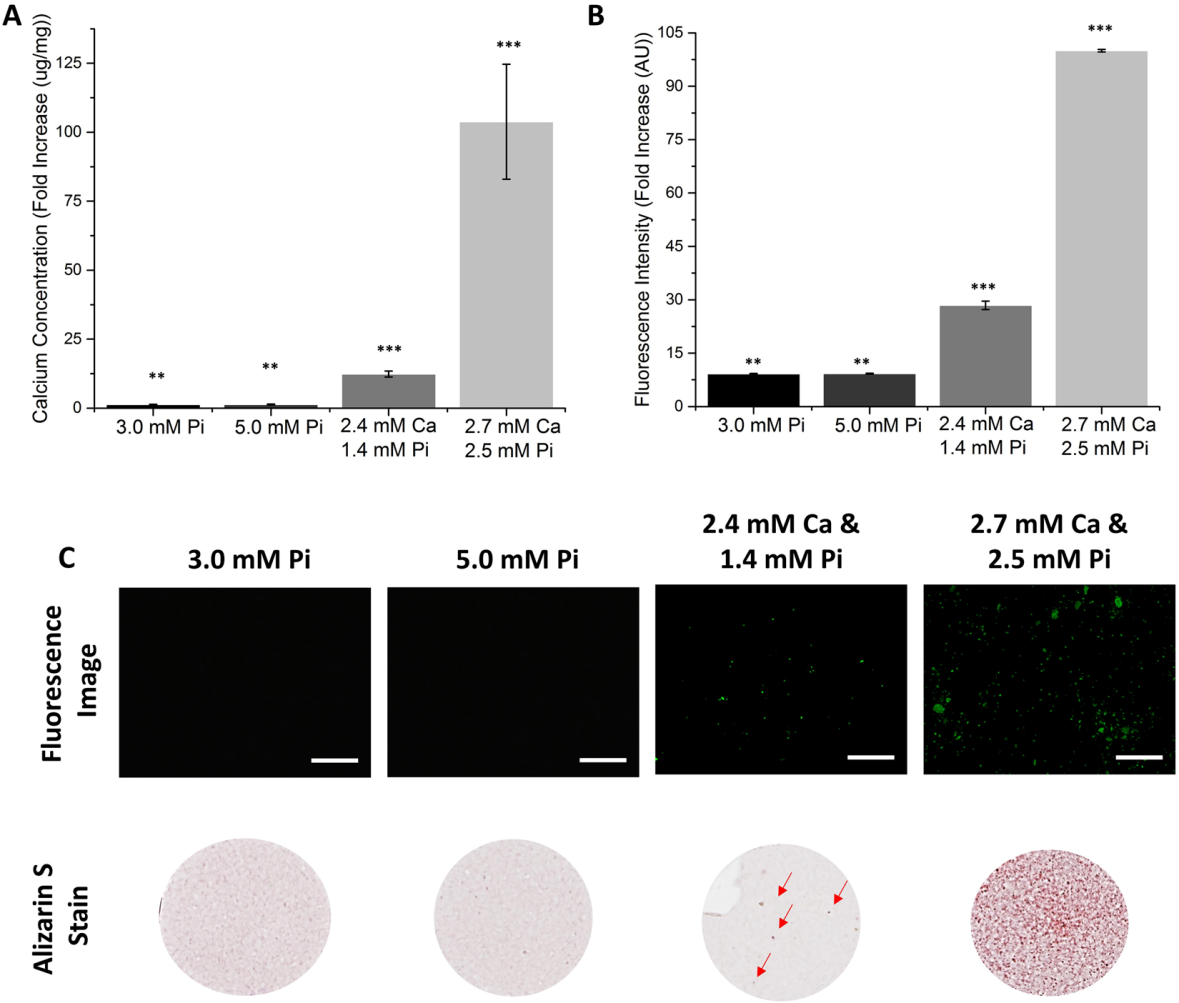
Determination of calcification in the MOVAS-1 cell line at various concentrations (3.0 mM Pi, 5.0 mM Pi, 2.4 mM Ca and 1.4 mM Pi, 2.7 mM Ca and 2.5 mM Pi). Monolayers of MOVAS-1 cells were grown to confluence (day 0) and then switched to calcification media. The monolayers were incubated in calcification media (3.0 mM Pi, 5.0 mM Pi, 2.4 mM Ca and 1.4 mM Pi, 2.7 mM Ca and 2.5 mM Pi) for 7 days, changing media on alternate days. (**A**) Calcium leaching assay: o-cresolphthalein complexone. (**B**) Fluorescence assay: cell monolayers were incubated with Fluorescein-BP (1 μM, 2 hours); NBF (10%, 15 minutes). (**C**) Fluorescence images and Alizarin S stain images: cell monolayers were either incubated with Fluorescein-BP (1 µM, 2 hours) and then fixed with NBF (10%, 15 minutes), or fixed with NBF (10%, 15 minutes) and then incubated with Alizarin S (2%, 500 μL, 10 minutes). Data shown in (**A**) and (**B**) are from at least 6 repeats and shown as the mean ± S.E.M., *P < 0.05, **P < 0.01, ***P < 0.001 compared to control, n = 6. Images shown in (**C**) are representative of at least 3 independent experiments yielding comparable results. Arrows indicate the location of calcium deposits within the well. Scale bars = 250 μm.

To probe the range of conditions under which the Fluorescein-BP probe **1** can be used, and compare its efficacy with the hydrochloric acid-based calcium leaching assay and Alizarin S stain, a further series of calcification assays were conducted using the MOVAS-1 cell line and primary mouse VSMCs. Monolayers of MOVAS-1 cells were incubated in both phosphate-enriched (3.0 mM Pi; 5.0 mM Pi), and phosphate and calcium-enriched (2.4 mM Ca and 1.4 mM Pi; 2.7 mM Ca and 2.5 mM Pi), growth media. Both the hydrochloric acid-based calcium leaching assay (4-fold, *p* < 0.001) (Fig. 3A) and the Fluorescein-BP **1** fluorescence assay (4-fold, *p* < 0.001) (Fig. 3B) show significant levels of calcium deposition in the presence of calcium-enriched media. The fluorescence assay (LOD 0.0065 µmol/L, SI Fig. 2) is able to detect the presence of HAP when using media enriched with only phosphate in comparison to the calcium leaching assay (LOD 225 µmol/L)^31^ demonstrating the greater sensitivity of Fluorescein-BP **1**. A comparison of the fluorescence images obtained for well plates with images of Alizarin S staining of equivalent plates (Fig. 3C), shows that both fail to detect calcification in media only enriched with phosphate. However, when cells are incubated in the presence of both calcium and phosphate, the fluorescent probe is able to detect the deposition of microcrystalline HAP with greater sensitivity than the Alizarin S stain. This is particularly notable when the incubation media have been enriched with 2.4 mM Ca and 1.4 mM Pi, as commonly used in vascular calcification models^32^.

**Figure 4:**
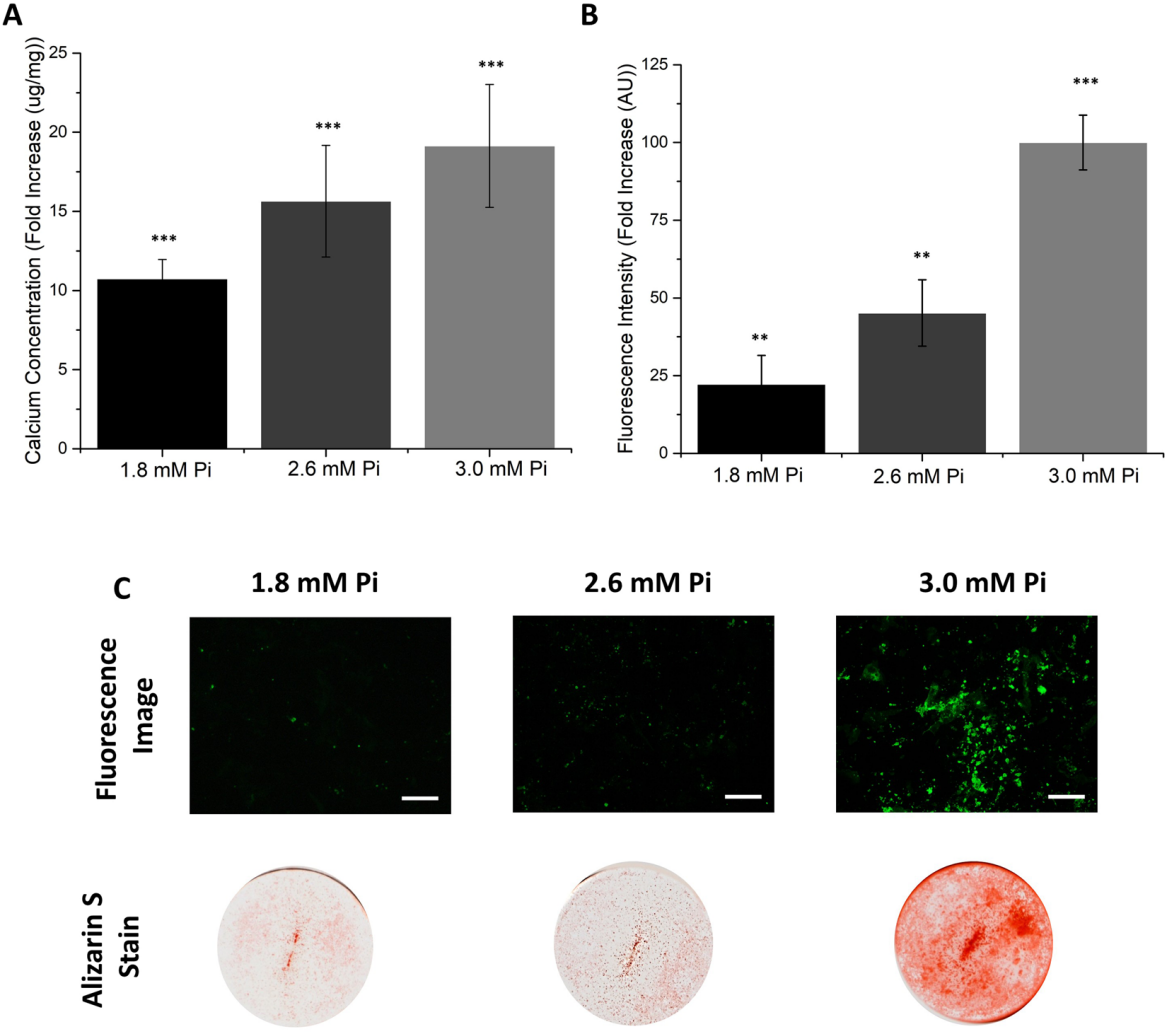
Determination of calcification in primary mouse VSMCs at various concentrations (1.8 mM Pi, 2.6 mM Pi, 3.0 mM Pi). Monolayers of primary mouse VSMCs were grown to confluence (day 0) and then switched to calcification media. The monolayers were incubated in calcification media (1.8 mM Pi, 2.6 mM Pi, 3.0 mM Pi) for 7 days, changing media on alternate days. (**A**) Calcium leaching assay: o-cresolphthalein complexone. (**B**) Fluorescence assay: cell monolayers were incubated with Fluorescein-BP (1 μM, 2 hours); NBF (10%, 15 minutes). (**C**) Fluorescence images and Alizarin S stain images: cell monolayers were either incubated with Fluorescein-BP (1 µM, 2 hours) and then fixed with NBF (10%, 15 minutes) or fixed with NBF (10%, 15 minutes) and then incubated with Alizarin S (2%, 500 μL, 10 minutes). Data shown in (**A**) and (**B**) are from at least 6 repeats and shown as the mean ± S.E.M., *P < 0.05, **P < 0.01, ***P < 0.001 compared to control, n = 6. Images shown in (**C**) are representative of at least 3 independent experiments yielding comparable results. *P < 0.05, **P < 0.01, ***P < 0.001 compared to control, n = 6. Scale bars = 250 μm.

When comparable studies were performed using primary mouse VSMCs (Fig. 4A–C), incubating the monolayers with increasing levels of phosphate to induce calcification (1.8 mM Pi, 2.6 mM Pi and 3.0 mM Pi), the fluorescent probe (5-fold, *p* < 0.001) was also shown to be more sensitive than either the Alizarin S or calcium leaching assays (1.2-fold, *p* < 0.001).

In further experiments, MOVAS-1 cells (SI Fig. 3) and primary mouse VSMCs (SI Fig. 4) were incubated under standard calcification conditions (2.4 mM Ca and 1.4 mM Pi) and the calcification on days 3, 5 and 7 was detected using Fluorescein-BP probe **1** (2.5-fold, *p* < 0.001 for MOVAS-1; 2.5-fold, *p* < 0.001 for VSMCs) and compared with calcium leaching using the hydrochloric acid-based assay (5-fold, *p* < 0.001 for MOVAS-1; 2.5-fold, *p* < 0.001 for VSMCs) and staining by Alizarin S. The fluorescent probe was shown to be more sensitive than both the other assays. Similarly, when MC3T3 cells (SI Fig. 5), an osteoblast derived cell line which is commonly used for studies in the field of bone and skeletal mineralisation^33,34^, were incubated with Fluorescein-BP, calcium mineralisation was readily detected (2-fold, *p* < 0.001).

### *Ex vivo* rat aorta and human tissue study

VSMC monolayer cultures are of only limited use in the study of calcification in CVD: they lack the architecture and matrix of normal vessels; they reach confluence over a short timeframe; they rapidly convert to a proliferative, secretory phenotype^35^; morphological variations have been seen at high passage numbers; and variations in the gene phenotype can also occur. Cultured aortae can provide complementary information that bridges the gap between traditional cell culture and animal models, under almost *in vivo* conditions^35,36^. Previous studies have cultured sectioned thoracic aortas dissected from mice and estimated the status of calcification under Pi stimulation at 2.6–3.0 mM Pi^36^; these phosphate concentrations correlate with myocardial infarction in human studies^36^.

**Figure 5:**
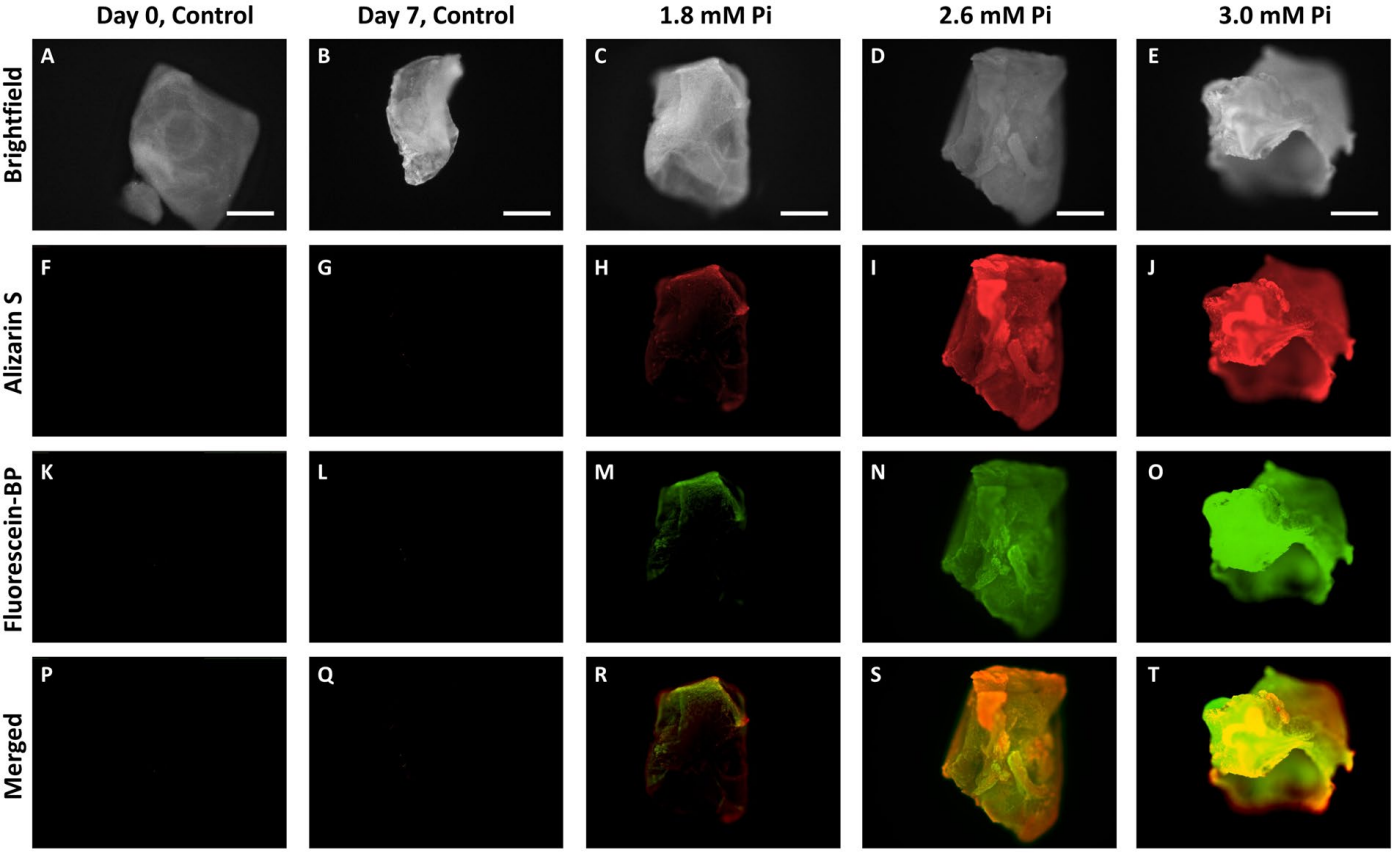
Determination of calcification in rat aorta incubated under different calcification conditions (1.8 mM Pi, 2.6 mM Pi and 3.0 mM Pi). Rat aortic rings were incubated for 2 days in fresh control media followed by calcification media (1.8 mM Pi, 2.6 mM Pi and 3.0 mM Pi) for 7 days. The rings were subsequently incubated with Fluorescein-BP (1 µM, 2 hours), fixed with NBF (10%, 15 minutes), permeabilised in KOH (1%, 1 hour) and then incubated with Alizarin S (0.00005% Alizarin S in 1% KOH, 24 hours). Brightfield (**A**–**E**) Fluorescence (**K**–**O**) Alizarin S (**F**–**J**). Merged images (**P**–**T**) recorded using both Alizarin S and Fluorescein-BP channels. Images shown are representative of at least 3 independent experiments yielding comparable results. Scale bars = 250 µm.

*Ex vivo* aortic rings from rats were incubated under different calcification conditions (1.8 mM Pi, 2.6 mM Pi and 3.0 mM Pi) for 7 days and subsequently incubated with Fluorescein-BP **1** followed by Alizarin S (Fig. 5, SI Fig. 6). From both staining methods, it is apparent that with increasing calcification conditions there is an increase in signal in both the Alizarin S and Fluorescein channel. When the two channels are merged, it is clear that the Alizarin S and Fluorescein-BP stains colocalize in most areas. No fluorescence signal is observed when the rings are cultured with control media confirming that the Fluorescein-BP probe is selective for HAP.

**Figure 6:**
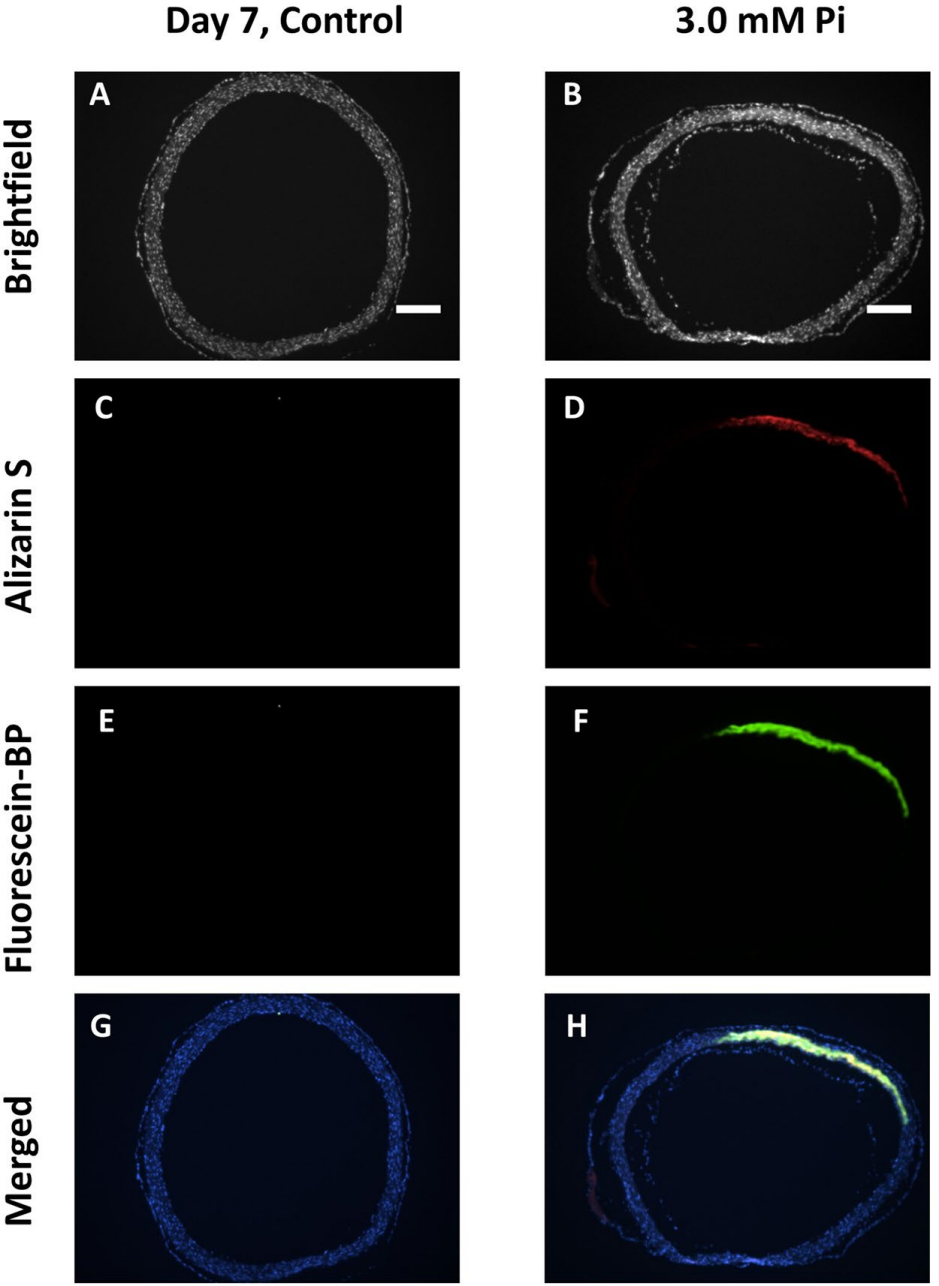
Determination of calcification in rat aortic ring sections. Rat aortic rings were incubated for 2 days in fresh control media followed by calcification media 3.0 mM Pi) for 7 days and fixed with NBF (10%, 15 minutes). Rings were cryosectioned into 5 μm sections. Sections were incubated with Fluorescein-BP (1 µM, 2 hours), permeabilised in KOH (1%, 1 hour), incubated with Alizarin S (0.00005% Alizarin S in 1% KOH, 24 hours), followed by DAPI (500 nM, 5 minutes). Brightfield (**A**,**B**) Fluorescence (**E**,**F**) Alizarin S (**C**,**D**). Merged images (**G**,**H**) recorded using Alizarin S, Fluorescein-BP and DAPI channels. Images shown are representative of at least 3 independent experiments yielding comparable results. Scale bars = 250 µm.

Next, a more detailed analysis of calcification in aortic rings was conducted using 5 µm sections (Fig. 6). Calcified rat aortic rings were fixed and cryosectioned on day 7; the sections were then incubated with Fluorescein-BP probe **1** followed by Alizarin S. Fluorescent microscopy of these sections confirms differences in calcification between the control and 3.0 mM Pi specimens. This also allows the location of the calcification to be determined; which in this rat model occurs in the medial layer of the aorta^35^.

**Figure 7:**
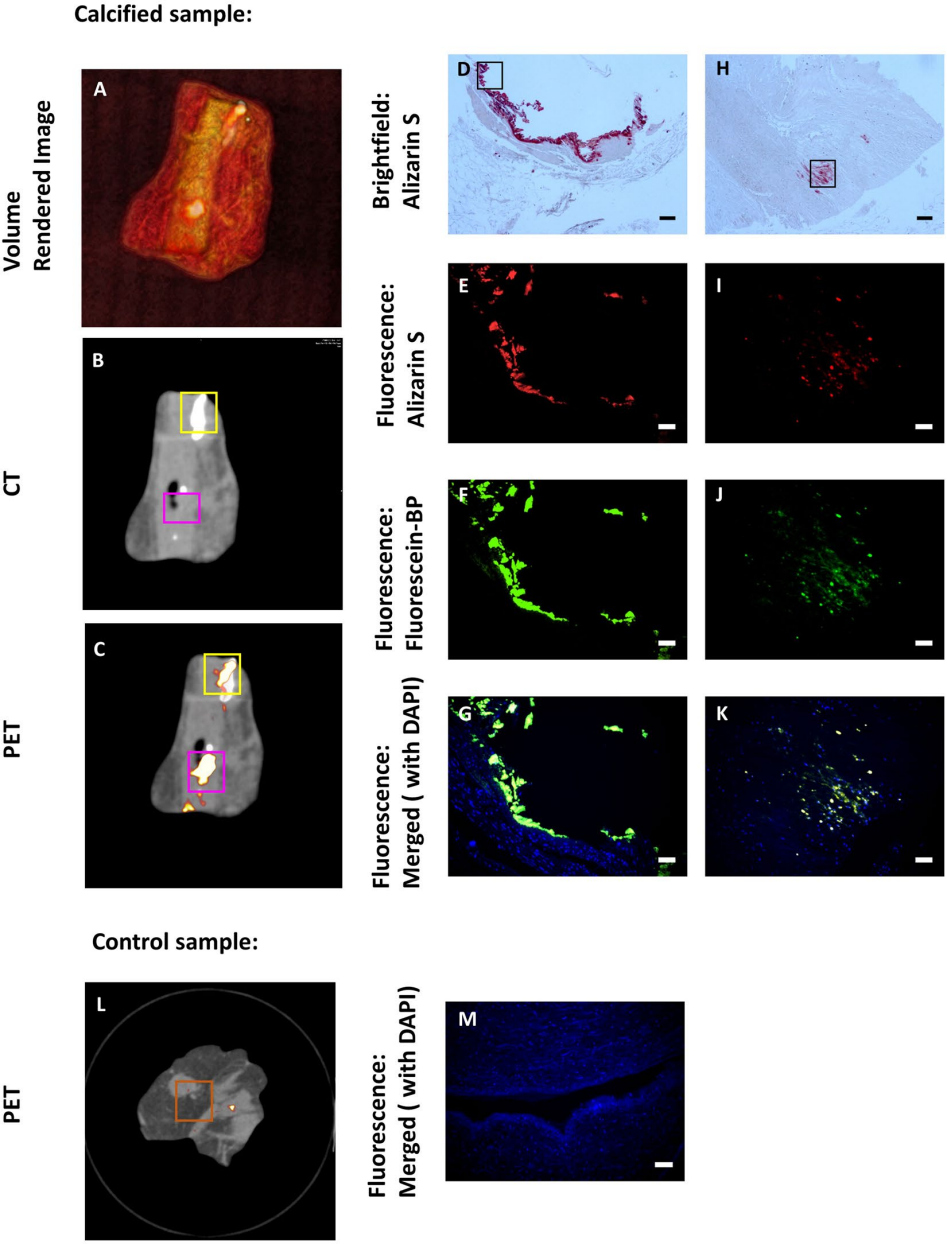
Determination of calcification in human vascular tissue (calcified and control). (**A**) Volume rendered image; (**B**) CT image; (**C**) PET image, with NaF tracer, of vascular tissue. Yellow, purple and orange boxes indicate the areas selected for tissue sectioning where Brightfield and Fluorescence images of calcification were taken. (**D**–**G**) Brightfield and Fluorescence (Alizarin S, Fluorescein-BP, DAPI) images of tissue in the yellow box which show high CT activity. (**H**–**K**) Brightfield and Fluorescence (Alizarin S, Fluorescein-BP, DAPI) images of tissue in the purple box which show high PET activity. Sections were incubated with Fluorescein-BP (1 μM, 2 hours), Alizarin S (2%, 10 minutes) and DAPI (500 nM, 5 minutes) and subsequently imaged. Black boxes indicate region of Brightfield image where Fluorescence images were taken. Control sample, PET image (**L**) and Fluorescence (Alizarin S, Fluorescein-BP and DAPI, **M**). Scale bars = 2.0 mm for Brightfield and 100 µm for Fluorescence.

PET/CT imaging using Na^18^F has been reported as a novel tool for vascular disease diagnosis^9,37^. Na^18^F is able to identify areas of micro-calcification that are associated with unstable plaque phenotype and which are beyond the resolution of CT. Indeed Na^18^F PET is currently being explored as a method for detecting high risk coronary plaques and improving cardiovascular risk prediction (clinicaltrials.gov NCT02278211)^9,37,38^. We next compared Fluorescein-BP binding to the detection of vascular calcification by computed tomography and Na^18^F PET in 8 samples of human arterial tissues (7 affected by calcific atherosclerotic disease and 1 control tissue which was free of atherosclerosis). All the tissue samples were assessed initially using PET/CT scans followed by sectioning of the tissue as required to allow appropriate fluorescence images to be obtained (as depicted for a representative sample in Fig. 7, additional examples shown in SI Fig. 7). PET/CT imaging (Fig. 7A–C) showed both areas of macrocalcification, as detected by CT, and areas of microcalcification, as detected by PET using a Na^18^F tracer. Samples from both areas were sectioned and stained (Fig. 7D–K). The images show that Fluorescein-BP probe **1** binds to both macro- and micro-calcification and colocalizes with Alizarin S staining (Fig. 7D,E). The control tissue sample (Fig. 7L,M), where there was no clinical history of coronary disease, showed neither PET/CT signal nor fluorescence staining.

## Discussion

Here, we demonstrate that Fluorescein-BP is a highly sensitivity and specific marker of HAP in vascular tissue. Fluorescein-BP has been compared to the current gold standard techniques for identifying calcification, the Alizarin S and HCl decalcification assays, and in both instances shows greater sensitivity and selectivity for HAP. Moreover we have confirmed its binding to HAP in both *in vitro* cell models of vascular and bone calcification, *ex vivo* rat aorta tissue and human coronary atherosclerotic plaques. Given that its component parts are already FDA approved, Fluorescein-BP holds great translational potential for the detection of crystalline HAP in humans.

Vascular mineralisation in atherosclerotic plaques contains high concentrations of crystalline HAP^39^. Within the arterial walls, extracellular vesicles containing HAP are extruded from lipid-rich macrophages and apoptotic smooth muscle cells which increase the plaque structural stress and risk of fibroathermatous cap fracture^40^. The inflammatory milieu driving the production of HAP deposition is associated with a greater propensity to destabilise plaques and results in clinical cardiovascular events^41,42^. Recent clinical trials have focused attention on the vital role that early calcification plays in carotid, aortic aneurysmal and coronary artery disease and hence there is a clinical imperative to better understand the mechanisms governing early calcification in the vascular wall^41–43^. The ability to differentiate calcium derivatives in regions of calcification, specifically by detecting HAP, will facilitate the dichotomisation of unstable and stable cardiovascular phenotypes in future clinical trials.

The detection of HAP deposition has been a field that has gained a lot of attention in the past 20 years^1,44–46^. Histological stains such as Alizarin S and von Kossa are widely used to study calcium deposits but their effectiveness has been disputed since their binding modes are somewhat indiscriminate^45^. Additionally, calcein is a routinely used tool for imaging calcium, however, not only does it bind across a range of calcium species, but it is also non-specific for calcium and interacts with a range of other metals including aluminium and zinc^47,48^. Another recently reported probe used in animal based models of both bone and vascular disease is Osteosense (680 and 750), a commercially available, near infrared (NIR) probe conjugated to a bisphosphonate^49–51^. However, the benefits offered by a NIR probe for *in vivo* studies, are off-set by the need for non-standard filters for plate readers and microscopes in laboratory based experiments which limited our capacity to directly compare the two probes.

Fluorescein-BP probe **1**, in comparison to Calcein and Osteosense, presents a more selective, sensitive and widely-applicable method of detecting calcification in vascular and bone models. In addition, the two low-cost reagents required for the synthesis of the probe are both commercially available, ensuring a wider user base for the new probe. Future *in vivo* studies might be enabled by intravascular administration of probe **1** using catheters fitted with a confocal endomicroscope probe, thus negating the need for conjugation to a costly NIR dye.

## Conclusions

Fluorescein-BP probe **1** gives a dramatically improved signal output over more conventional imaging methods such as Alizarin S and von Kossa stains for the detection of calcium phosphate in a range of *in vitro* studies. It has been shown to be mineral-specific giving a markedly increased signal in the presence of HAP over other calcium species, and can be used in both condition-specific and temporal studies of calcification in cell-based models of CVD and bone disease. Staining with probe **1** is rapid, allowing high-throughput assays in multi-well format which are not possible with comparable assays such as the hydrochloric acid-based quantification method typically used in the field. When applied to *ex vivo* aortic sections and to human tissue presenting both macro- and micro-calcification, Fluorescein-BP **1** shows binding to both types of vascular calcification which are not readily detectable using one stand-alone technique. The employment of this probe may enable the elucidation of key mechanisms underpinning these pathological processes.

## Methods

Unless otherwise noted, starting materials and reagents were obtained from Sigma-Aldrich and were used without further purification. Saturated aqueous solutions of inorganic salts are represented as (volume, sat. aq.). Semi-preparative RP-HPLC was performed using a Waters 600E pump, a Waters 486 tuneable absorbance detector controlled by Water Empower software which was equipped with a Phenomenex Luna C18(2), 5 µm particle size, 250 × 21.2 mm column at a flow rate of 18 mL min^−1^.

^1^H and ^13^C NMR spectra were obtained on Bruker AVA600 instrument at the stated frequency using TMS as a reference and residual solvent as an internal standard. Infra-red spectra were recorded neat on a Shimadzu IRAffinity-1. Electrospray (ESI) mass spectra were obtained on a Bruker micrOTOF II instrument. UV/VIS absorption spectra were measured using a Shimadzu UV-1800 spectrometer. Melting points were determined on a Gallenkamp Electrothermal Melting Point apparatus and are uncorrected. Fluorescence data was obtained using SPEX Fluoromax-3.

### Fluorescein-BP conjugate (1)

Sodium alendronate (34 mg, 1 mmol, 5 eq; Alfa Aesar) was dissolved in NaHCO_3_ (1 mL, sat. aq.). Fluorescein (5/6) NHS ester (10 mg, 0.2 mmol, 1 eq; Thermo Fisher) dissolved in DMF (0.1 mL) was added to the solution and the reaction mixture was stirred for 2 days at room temperature in the dark. The solution was acidified with TFA (10% v/v aq.) and purified by RP HPLC [isocratic 80/20 H_2_O (+0.1% v/v TFA)/MeCN (+0.1% v/v TFA)] to yield the fluorescein-BP conjugate as an orange solid (34 mg, 50%).

λ_abs_/λ_em_ 491/515; Φ 0.83; ε 41,400 M^−1^cm^−1^; MP 240 °C; IR (solid, cm^−1^) 1700 (C=O), 1635 (C=O), 1538 (N-H), 1271 (P=O), 1116 (C-N); ^1^H NMR (601 MHz, DMSO-*d*_6_) δ 10.13 (2 H, br s, O*H isomer 1* + 2) 8.85 (0.6 H, t, *J* = 5.7 Hz, N*H isomer 1*), 8.72 (0.4 H, t, *J* = 5.9 Hz, N*H isomer* 2), 8.47 (0.6 H, d, *J* = 1.5 Hz, Ar*H isomer 1*), 8.26 (0.6 H, dd, *J* = 8.0, 1.5 Hz, Ar*H isomer 1*), 8.19 (0.4 H, dd, *J* = 8.1, 1.4 Hz, Ar*H isomer* 2), 8.07 (0.4 H, d, *J* = 8.1 Hz, Ar*H isomer* 2), 7.70 (0.4 H, s, Ar*H isomer 2*), 7.37 (0.6 H, d, *J* = 8.0 Hz, Ar*H isomer 1*), 6.69 (2 H, dd, *J* = 5.3, 2.3 Hz, Ar*H isomer 1* + *2*), 6.63 – 6.53 (4 H, m, Ar*H isomer 1* + *2)* 3.30 (1.2 H, q, *J* = 6.5 Hz, NC*H*_*2*_ *isomer 1*), 3.17 (0.8 H, q, *J* = 6.4 Hz, NC*H*_*2*_ *isomer 2*), 2.01-1.70 (4 H, m, C*H*_*2*_C*H*_*2*_ *isomer 1* + *2*); ^13^C NMR (151 MHz, DMSO-*d*_6_) δ 168.69 (C=O), 168.55 (C=O), 164.86 (C=O), 164.67 (C=O), 160.06 (2 × Ar C), 155.02 (Ar C), 153.17 (Ar C), 152.32 (2 × Ar C), 152.30 (2 × Ar C), 141.35 (Ar C), 136.96 (Ar C), 135.16 (Ar CH), 129.85 (ArCH), 129.74 (2 × ArCH), 129.66 (2 x ArCH), 128.55 (Ar C), 126.92 (Ar C), 125.27 (Ar CH), 124.66 (Ar CH), 123.72 (Ar CH), 122.73 (Ar CH), 113.21 (2 x Ar CH), 113.16 (2 x Ar CH), 109.67 (2 × Ar C), 109.60 (2 × Ar C), 102.73 (2 × Ar CH), 83.75 (C), 83.72 (C), 72.60 (t, ^1^*J*_CP_ = 148 Hz, C), 41.99 (t, ^2^*J*_CP_ = 21 Hz, CH_2_), 41.65 (t, ^2^*J*_CP_ = 21 Hz, CH_2_), 31.13 (CH_2_), 24.06 (CH_2_); *m/z* (ESI+, H_2_O:MeOH): 630 ([M+Na]^+^, 10%), 608 ([M+H]^+^, 100); ^31^P NMR (202 MHz, DMSO-*d*_6_) δ 20.21.

### Fluorescein-BP and Alizarin S incorporation in Calcium minerals

Aqueous suspensions of calcium species (HAP, CaOx, CaPi, CaPyr, Sigma and Fisher Scientific) (50 mg) were incubated with Fluorescein-BP **1** (5 µM) for 2 hours. Unbound probe was removed by centrifugation of the suspension at 670 *g* for 5 minutes, the supernatant was removed and the precipitated solid was resuspended in water with continuous agitation for 15 minutes. This process of centrifugation and resuspension was repeated three times, until there was no further fluorescence in the supernatant after centrifugation. Fluorescence analysis of the combined aqueous extracts was performed using a Synergy HT Multi Mode Microplate reader at 488 nm.

### VSMC isolation and culture

Primary aortic VSMCs were isolated from five week old C57BL/6 mice (Charles River Laboratories) as previously described^1,52,53^. Mice were euthanized by cervical dislocation, the aorta was then dissected and the adventitia removed. After washing with Hanks’ balanced salt solution (HBSS; Life Technologies), the aorta was cut longitudinally to expose the endothelial layer. Eight aortae were pooled together and incubated for 10 minutes at 37 °C in 1 mg/mL trypsin (Life Technologies) to remove any remaining adventitia and endothelium. Aortae were washed and incubated overnight at 37 °C in VSMC growth medium containing Minimum Essential Medium Eagle alpha modification (α-MEM; Life Technologies), 10% foetal bovine serum (FBS; Life Technologies) and 1% gentamicin (Life Technologies) in a humidified 5% CO_2_ incubator. Tissues were then washed and incubated in 425 UI/mL collagenase type II (Worthington Biochemical Corporation) for 4 hours at 37 °C. The resulting cell suspension was centrifuged at 320 *g* for 5 minutes. VSMC pellets were resuspended in culture medium and cultured for two passages in T25 tissue culture flasks (Corning) coated with 0.25 μg/cm^2^ laminin (Sigma) to promote maintenance of the contractile differentiation state^54^.

### MOVAS-1 culture

MOVAS-1 cells (ATCC) were suspended in growth media consisting of Dulbecco’s Modified Eagle Media (DMEM)-Formula 12 (Life Technologies) supplemented with 10% FBS and 1% gentamicin and cultured at 37 °C, in the presence of 5% CO_2_. Before experimentation, MOVAS-1 were expanded in growth media and cells were then used for experiments.

### Induction of calcification in MOVAS-1 and VSMCs

Calcification was introduced as previously described^1,32,53^. In brief, for MOVAS-1, cells were grown to confluence (day 0) and switched to calcification medium, which was prepared by adding 1 M inorganic phosphate (mixture of NaH_2_PO_4_ and Na_2_HPO_4_, pH = 7.4) and 1 M CaCl_2_ to reach a final concentrations of 3.0 mM Pi, 5.0 mM Pi, 2.4 mM Ca and 1.4 mM Pi or 2.7 mM Ca and 2.5 mM Pi. For VSMCs, cells were grown to confluence (day 0) and switched to calcification medium, which was prepared by adding 1 M inorganic phosphate to reach a final concentrations of 1.8 mM Pi, 2.6 mM Pi and 3.0 mM Pi. Cells were incubated for 7 days in 95% air/5% CO_2_, changing media on alternate days.

### Fluorescence staining of fixed cells using Fluorescein-BP and CellMask Orange Plasma Membrane Stain

MOVAS-1 cells were grown on glass coverslips and calcified as described above. After incubation with Fluorescein-BP probe **1** (1 μM) for 2 hours, the media was changed, the monolayer was washed twice with HBSS and fresh HBSS containing 500 nM CellMask Orange Plasma Membrane Stain (Thermo Fisher) was added for 10 minutes. The monolayer was washed with phosphate buffered saline (PBS, 2×) and water (1×), fixed with 10% neutral buffered formalin (NBF) for 15 minutes and washed with PBS (2×) and water (1×). The cell monolayers were permeabilised with Triton X-100 (0.05%, 3×) for 5 minutes and incubated with DAPI (300 nM; Life Technologies) for 5 minutes. Excess DAPI was removed by washing with water (1×). Glass coverslips were mounted onto slides with Prolong Gold Anti-Fade Reagent (Life Technologies). Fluorescence signal was detected under a Zeiss Axiovert 25 inverted microscope and/or Zeiss Confocal LSM 710 at 488 nm (Fluorescein-BP), 554 nm (CellMask Orange) and 350 nm (DAPI).

### Raman microscopy

MOVAS-1 were grown and calcified (2.4 mM Ca and 1.4 mM Pi) on CaF_2_ slides. After 7 days, the monolayer was fixed with 10% NBF for 15 minutes. Raman imaging was carried out using an InVia Renishaw Microscope with laser excitation at 785 nm and a 50, N.A. 0.75 objective. The total data acquisition was performed during 30 s for spectra with a 50% laser power using the WiRE software. All of the spectra acquired were background subtracted using a background correction algorithm in the WiRE software.

### Fluorescence microscopy

MOVAS-1 and VSMCs were grown on plates and calcified as described above. Media was removed and the cell monolayer was subsequently washed with HBSS then incubated with DMEM/F12 (MOVAS-1) and α-MEM (VSMCs) containing Fluorescein-BP probe **1** (1 μM) for 2 hours. The media was then extracted and the cell monolayer was washed twice with HBSS, fixed with 10% NBF for 15 minutes and washed with PBS (PBS, 2×) and water (1×). Fluorescence images were captured using a Leica DMBL-2 upright fluorescent microscope and on Synergy HT Multi Mode Microplate reader at 488 nm (Fluorescein-BP).

### Calcium leaching assay

Calcium deposition was quantified as previously described^32,52^. Briefly, cells were rinsed with phosphate buffered saline (PBS) and decalcified with 0.6 N HCl at room temperature for 24 hours. Free calcium was determined calorimetrically by a stable interaction with *o*-cresolphthalein complexone using a commercially available kit (Randox Laboratories Ltd.) and corrected for total protein concentration (Bio-Rad Laboratories Ltd).

### Alizarin S staining of cell monolayers

Cells were grown in plates and calcified as described above. Media was removed and the cell monolayer was subsequently washed with HBSS. Cells were fixed in 10% NBF for 15 minutes before washing twice with PBS. The cells were then stained with Alizarin S (2%, pH 4.2; 500 µL) for 10 minutes at rt. The supernatant was discarded and the cell monolayer was washed with water (3×) and then imaged at 530 nm (Alizarin S).

### *Ex vivo* aorta isolation and culture

Aortic rings were dissected from eight week old Fischer male rats. Rats were euthanized by cervical dislocation, the aorta was then dissected and the adventitia removed. After washing with HBSS the aortae were cut into ~4 mm thick rings and cultured in α-MEM supplemented with 10% FBS and 1% gentamicin at 37 °C in 95% air/5% CO_2_. After 1 day, the media was changed to calcifying media (1.8 mM Pi, 2.6 mM Pi, 3.0 mM Pi) for 7 days, changing media on alternate days. On day 7, aortas were incubated with Fluorescein-BP (1 µM) for 2 hours. Aortas were washed with PBS (2×) and fixed with 10% NBF for 15 minutes. Residual NBF was removed by washing with PBS (2×) and water (1×). For Alizarin S staining, the fixed aortas were permeabilised with 1% KOH for 1 hour followed by overnight staining with 0.00005% Alizarin S in 1% KOH. Residual Alizarin S was removed by washing with water (3×). Rings were then imaged under a Zeiss Axiovert 25 inverted microscope at 488 nm (Fluorescein-BP) and 530 nm (Alizarin S).

### Aortic sections

Aortic rings were dissected and calcified following the standard protocol. Once calcified, the rings were fixed in 10% NBF for 15 minutes and then cryosectioned into 5 μm thick sections. Sections were then imaged under a Leica DMBL-2 upright fluorescent microscope at 488 nm (Fluorescein-BP), 530 nm (Alizarin S) and 350 nm (DAPI).

### Human tissue

Atherosclerotic layers of left main coronary arteries were analysed from a range of patients, both male and female between the age of 48–71, for which main cause of death was either haemopericardium or ischaemic and hypertensive heart disease. Upon approval, the tissue was frozen at −80 °C.

### PET/CT analysis of human tissue

Thawed non-decalcified coronary artery specimens were incubated for 20 minutes in ^18^F-sodium fluoride 100 kBq/mL solution (10.5 MBq 18F-NaF in 99.5 mL 0.9% NaCl). Samples were twice washed in 10 mL 0.9% NaCl for 5 minutes to remove unbound ^18^F-fluoride. Coronary artery specimens were scanned using high-resolution micro-positron emission tomography and non-contrast computed tomography [50 kV p tube voltage, 300 ms exposure time] (Mediso nanoScan PET/CT). After whole specimen imaging, the coronary arteries were fixed in 10% buffered formaldehyde before being dehydrated, embedded in paraffin wax and sectioned (5 μm thickness).

### Alizarin S Staining of human tissue

Sections were de-waxed in xylene and stained with 2% Alizarin S for 5 minutes, washed three times with water and dehydrated to visualize calcium deposition. Images were obtained using a Nikon Ni1 Brightfield microscope.

### Fluorescein-BP and Alizarin S Staining of human tissue

Sections were de-waxed in xylene and incubated with Fluorescein-BP probe **1**(1 µM) for 2 hours, washed in water (2×) followed by incubation with 2% Alizarin S (250 µL) for 5 minutes. Sections were washed in water (3×) and subsequently incubated with DAPI (500 nM) for 5 minutes. Sections were washed with water (1×) and then mounted using ProLong Gold Antifade. Fluorescence signal was detected using a Leica DMRB fluorescence microscope at 488 nm (Fluorescein-BP), 530 nm (Alizarin S) and 350 nm (DAPI).

### Statistical analysis

Two-sample Student’s *t*-test was used to analyse the significance between data sets. Data are presented as mean ± standard error of the mean (S.E.M.). All statistical analysis was performed using Minitab 17 (Minitab Inc.). *p* < 0.05 was considered to be significant, and *p-*values are represented as: *p < 0.05; **p < 0.01; ***p < 0.001.

### Ethical approval

All animal experiments were approved by The Roslin Institute’s Animal Users Committee and the animals were maintained in accordance with Home Office guidelines for the care and use of laboratory animals. Atherosclerotic layers of left main coronary arteries were obtained from victims of sudden death at autopsy with written informed relative authorisation from the next of kin. Ethical approval was granted by National Health Service Research Ethics Committee South East Scotland (14/SS/1090). The retention, storage and use of tissue sections were compliant with the United Kingdom Human Tissue Act of 2004 and in accordance with the relevant guidelines and regulations approved by National Health Service Research Ethics Committee South East Scotland (14/SS/1090).

## Electronic supplementary material

Supplementary Information

## Data Availability

All data generated and analysed during this study are available as Supplementary Data online. Primary data files are available at 10.7488/ds/2467.
