# Supplementary material for: A novel fluorescein-bisphosphonate based diagnostic tool for the detection of hydroxyapatite in both cell and tissue models
Source: Sci Rep. 2018 Nov 26;8:17360. doi: 10.1038/s41598-018-35454-9 (PMC6255785; doi:10.1038/s41598-018-35454-9)
Supplement: Supplementary file 1 — Supplementary Information [file 41598_2018_35454_MOESM1_ESM.pdf]

A novel fluorescein-bisphosphonate based diagnostic tool for the detection of hydroxyapatite in both cell and tissue models

Alisia M. Sim[1,2,3], Nabil A. Rashdan[2], Lin Cui[2], Alastair J. Moss[3], Fabio Nudelman[1], Marc R. Dweck[3], Vicky E. MacRae[2] and Alison N. Hulme[1*]

[1] EaStCHEM School of Chemistry, University of Edinburgh, David Brewster Road, Edinburgh, EH9 3FJ, UK;
[2] The Roslin Institute, Royal (Dick) School of Veterinary Studies, University of Edinburgh, Easter Bush, Midlothian EH25 9RG, UK;
[3] Centre for Cardiovascular Science, University of Edinburgh, Edinburgh, EH16 4UU, UK

*Corresponding author email: Alison.Hulme@ed.ac.uk

Page No **Contents**

Page No	Contents
S1	Synthesis of fluorescein-5-amino-pentan-1-ol conjugate
S3	Incubation of fluorescein-OH conjugate with a calcified MOVAS-1 cell monolayer
S3	Calibration curve for the Fluorescein-BP (**1**) conjugate
S4	Calcification in MOVAS-1
S5	Calcification in VSMCs
S6	Calcification in MC3T3
S7	*Ex vivo* Aorta controls
S8	Human tissue samples
S8	References

Synthesis of fluorescein-5-amino-1-pentanol conjugate

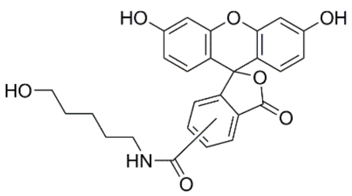

Prepared following the method of Gavin *et al.*.[1] 5-amino-1-pentanol (34 mg, 1 mmol, 5 eq) was dissolved in $NaHCO_3$ (1 mL, sat. aq.). Fluorescein (5/6) NHS ester (10 mg, 0.2 mmol, 1 eq) dissolved in DMF (0.1 mL) was added to the solution and the reaction mixture was stirred for 2 days at room temperature in the dark. The solution was acidified with TFA (10% v/v aq.) and purified by RP HPLC [isocratic 80/20 H_2O (+0.1% v/v TFA)/MeCN (+0.1% v/v TFA)] to yield the fluorescein-OH conjugate as an orange solid (5 mg, 34%).

λ_{abs}/λ_{em} 491/517; **Φ** 0.87; **ε** 38,400 $M^{-1}cm^{-1}$; **MP** 227 °C; **IR** (solid, cm^{-1}): 3410 (OH), 1624 (C=O), 1498 (N-H), 1106 (C-N); **^{1}H NMR** (601 MHz, DMSO-*d*$_6$) δ 10.11 (2H, br s, O*H isomer 1+2*), 8.80 (0.6H, t, *J* = 6.5 Hz, N*H isomer 1*), 8.66 (0.4H, d, *J* = 6.5 Hz, N*H isomer 2*), 8.46 (0.6H, d, *J* = 6.6 Hz, Ar*H isomer 1*), 8.25 (0.6H, t, *J* = 7.5 Hz, Ar*H isomer 1*), 8.17 (0.4H, t, *J* = 7.5 Hz, Ar*H isomer 2*), 8.08 (0.4H, t, *J* = 7.6 Hz, Ar*H isomer 2*), 7.67 (0.4H, d, *J* = 6.5 Hz, Ar*H isomer 2*), 7.37 (0.6H, t, *J* = 7.6 Hz, Ar*H isomer 1*), 6.70 (2H, br s, Ar*H isomer 1+2*), 6.63 – 6.52 (4H, m, Ar*H isomer 1+2),* 3.42-3.32 (2H, m, *CH$_2$*OH *isomer 1+2*), 3.32 (1.2H, m, NC*H$_2$ isomer 1*), 3.19 (0.8H, t, *J* = 6.8 Hz, NC*H$_2$ isomer 2)*, 1.56 (1.2H, m, C*H$_2$ isomer 1*), 1.49-1.21 (4.8H, m, 2 x C*H$_2$ isomer 1+2 + CH$_2$ isomer 2*); **^{13}C NMR** (126 MHz, DMSO-*d*$_6$) δ 168.69 (CO), 168.53 (CO), 164.95 (CO), 164.77 (CO), 160.09 (2 × Ar C), 155.04 (Ar C), 153.15 (Ar C), 152.31 (2 × Ar C), 141.31 (Ar C), 136.90 (Ar C), 135.15 (Ar CH), 129.85 (Ar CH), 129.72 (2 × Ar CH), 129.63 (2 × Ar CH), 128.59 (Ar C), 126.92 (Ar C), 125.32 (Ar CH), 124.68 (Ar CH), 123.68 (Ar CH), 122.67 (Ar CH), 113.23 (2 x Ar CH), 113.16 (2 x Ar CH), 109.66 (2 x Ar C), 109.59 (2 x Ar C), 102.75 (2 × Ar CH), 102.73 (2 × ArCH), 83.78 (C), 61.11 (CH_2), 61.03 (CH_2), 39.86 (CH_2), 32.71 (CH_2), 32.68 (CH_2), 29.35 (CH_2), 29.28 (CH_2), 23.51 (CH_2); ***m/z*** (ESI+, H_2O:MeOH): 558 (5%), 462 ($[M+H]^+$, 100).

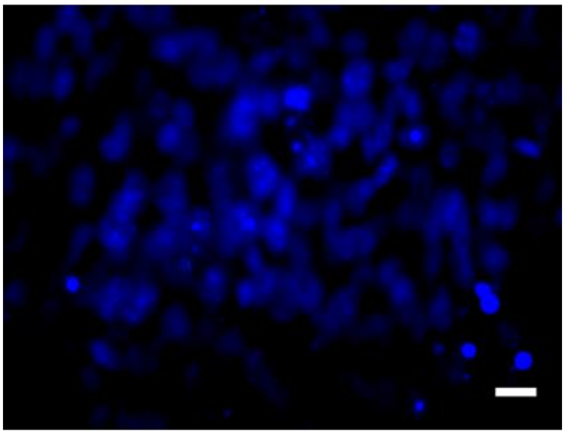

SI Figure 1: Fluorescence image of Calcified MOVAS-1 cells incubated with Fluorescein-OH. A monolayer of MOVAS-1 cells was grown to confluence (day 0) and then switched to calcification media (2.4 mM Ca and 1.4 mM Pi) for 7 days, changing media on alternate days. The cell monolayer was incubated with Fluorescein-OH (1 µM) for 2 hours, fixed with NBF (10%, 15 minutes) and mounted using ProLong Gold Antifade with DAPI. Scale bar = 100 µm.

Fluorescence calibration curve:

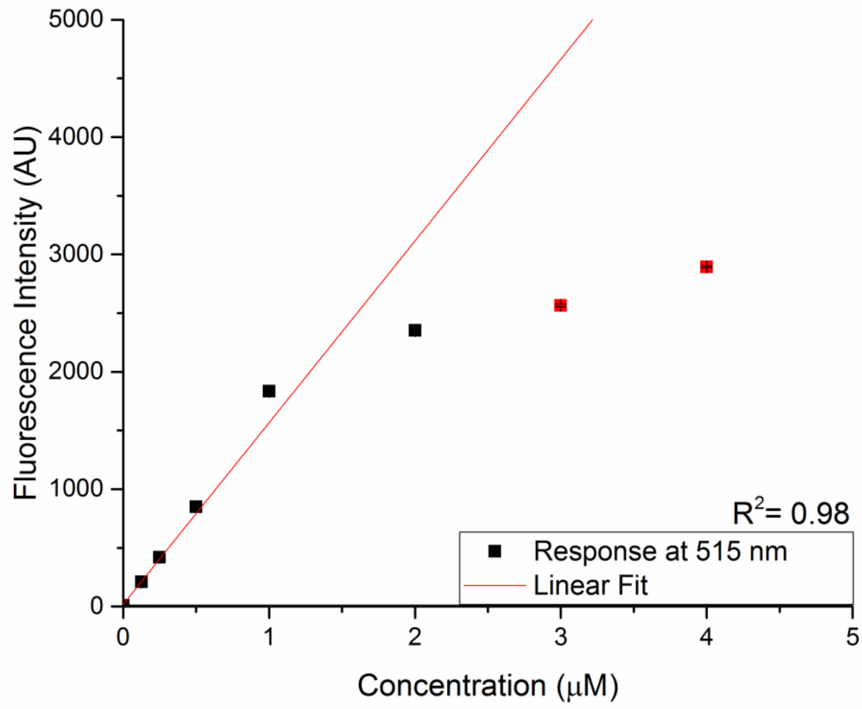

SI Figure 2: Calibration curve showing lowest concentration of detection of Fluorescein-BP (1). Data shown from 5 repeats and shown as the mean ± S.E.M.

The limit of detection (LOD) was calculated based on the following equation.

LOD = mean blank + 3*SD = 10.8 AU Corresponding to 0.0065 µM

MOVAS-1 and primary mouse VSMCs day study:

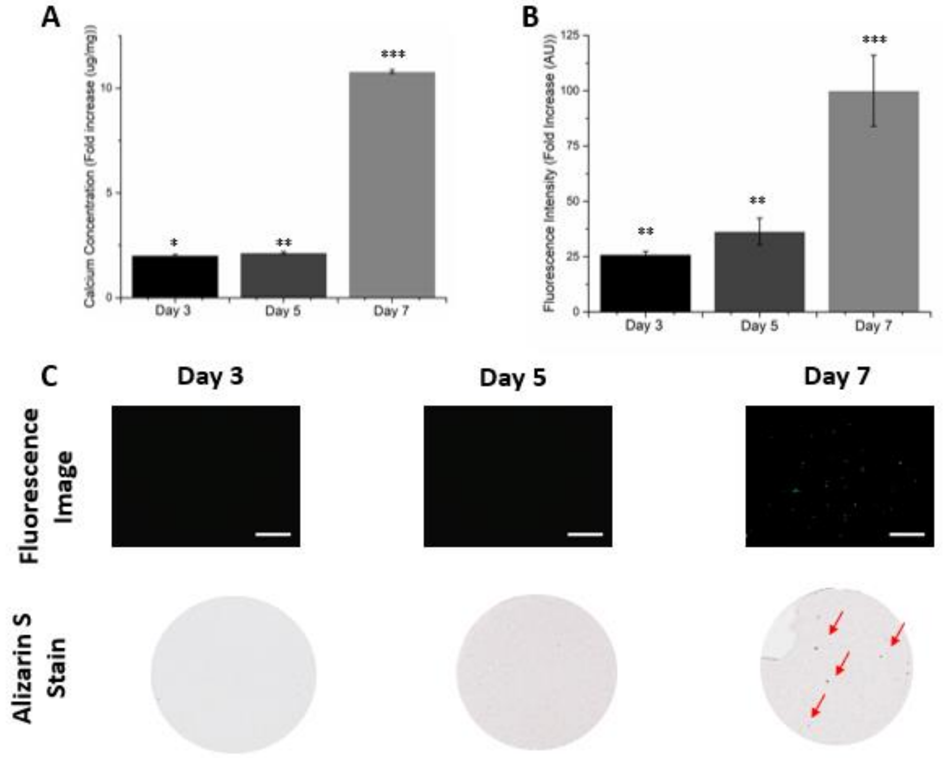

SI Figure 3: Determination of calcification in MOVAS-1 at various incubation days (3, 5 and 7). A monolayer of MOVAS-1 cells was grown to confluence (day 0) and then switched to calcification media (2.4 mM Ca and 1.4 mM Pi) for 3, 5, or 7 days, changing media on alternate days. (**A**) Calcium leaching assay: *o*-cresolphthalein complexone. (**B**) Fluorescence assay; cell monolayers were incubated with Fluorescein-BP (1 µM, 2 hours); NBF (10%, 15 minutes). (**C**) Fluorescence images and Alizarin S stain images: cell monolayers were either incubated with Fluorescein-BP (1 µM, 2 hours) and then fixed with NBF (10%, 15 minutes), or fixed with NBF (10%, 15 minutes) and then incubated with Alizarin S (2%, 500 µL, 10 minutes). Data shown in (**A**) and (**B**) are from at least 6 repeats and shown as the mean ± S.E.M., * $P < 0.05$, ** $P < 0.01$, *** $P < 0.001$ compared to control, n = 6. Images shown in (**C**) are representative of at least 3 independent experiments yielding comparable results. Scale bars = 250 µm.

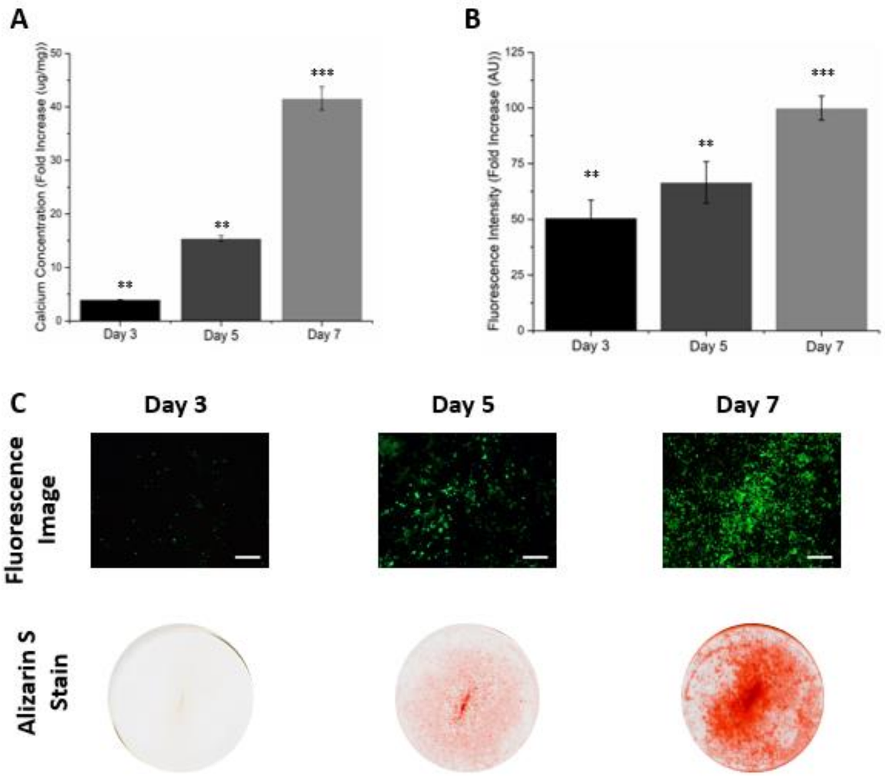

SI Figure 4: Determination of calcification in VSMCS at various incubation days (3, 5 and 7). A monolayer of VSMCS cells was grown to confluence (day 0) and then switched to calcification media (3.0 mM Pi) for 3, 5, or 7 days, changing media on alternate days. (**A**) Calcium leaching assay: *o*-cresolphthalein complexone. (**B**) Fluorescence assay: cell monolayers were incubated with Fluorescein-BP (1 µM, 2 hours); NBF (10%, 15 minutes). (**C**) Fluorescence images and Alizarin S stain images: cell monolayers were either incubated with Fluorescein-BP (1 µM, 2 hours) and then fixed with NBF (10%, 15 minutes), or fixed with NBF (10%, 15 minutes) and then incubated with Alizarin S (2%, 500 µL, 10 minutes). Data shown in (**A**) and (**B**) are from at least 6 repeats and shown as the mean ± S.E.M., * $P < 0.05$, ** $P < 0.01$, *** $P < 0.001$ compared to control, n = 6. Images shown in (**C**) are representative of at least 3 independent experiments yielding comparable results. Scale bars = 250 µm.

MC3T3 cell line

Cells were cultures in α-MEM (Life Technologies) supplemented with 10% FBS (Life Technologies) and 1% gentamicin (Life Technologies) at 37°C in 95% air/5% CO_2. Calcification was introduced as previously described.[2,3] In brief, cells were grown to confluence (day 0) and switched to calcification medium, which was prepared by adding 2.5 mM β-glycerophosphate (β-GP;) and 50 µg/mL ascorbic acid (AA). Cells were incubated for 7 days in 95% air/5% CO_2 and the medium was changed on alternate days.

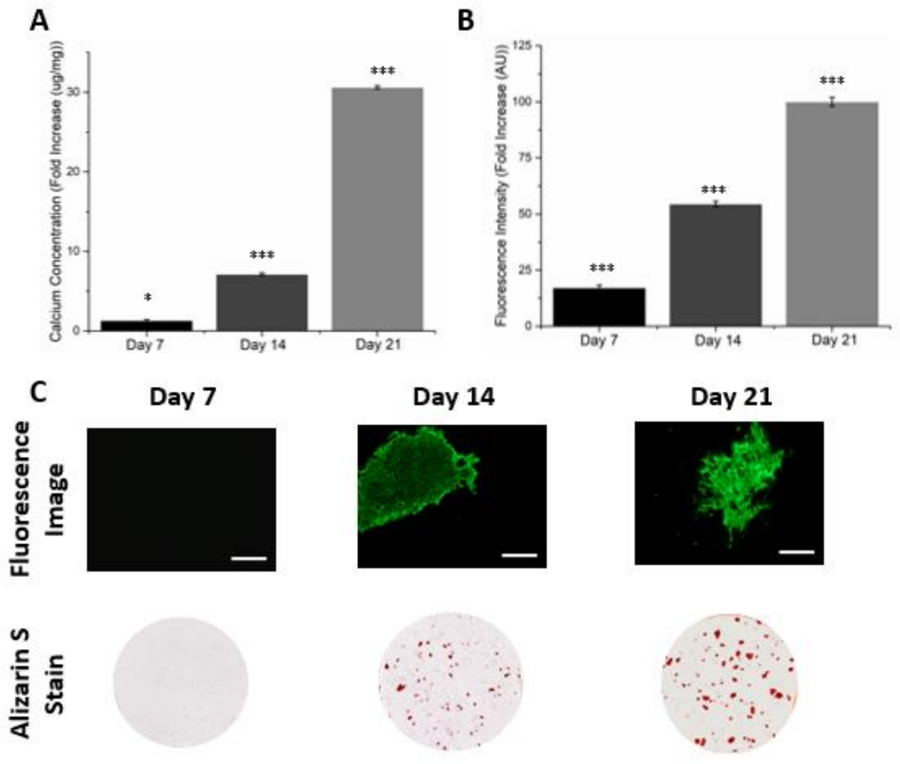

SI Figure 5: Determination of calcification in MC3T3 at various days incubation (7, 14 and 21). MC3T3 cells were grown to confluence (day 0) and switched to calcification media (2.5 mM β-GP and 50 µg/ml AA). Cells were incubated for 7, 14, or 21 days, changing media on alternate days. (**A**) Calcium leaching assay: *o*-cresolphthalein complexone. (**B**) Fluorescence assay: cell monolayers were incubated with Fluorescein-BP (1 µM, 2 hours); NBF (10%, 15 minutes). (**C**) Fluorescence images and Alizarin S stain images: cell monolayers were either incubated with Fluorescein-BP (1 µM, 2 hours) and then fixed with NBF (10%, 15 minutes), or fixed with NBF (10%, 15 minutes) and then incubated with Alizarin S (2%, 500 µL, 10 minutes). Data shown in (**A**) and (**B**) are from at least 6 repeats and shown as the mean ± S.E.M., * $P < 0.05$, ** $P < 0.01$, *** $P < 0.001$ compared to control, n = 6. Images shown in (**C**) are representative of at least 3 independent experiments yielding comparable results. Scale bars = 250 µm.

Ex vivo Aorta controls

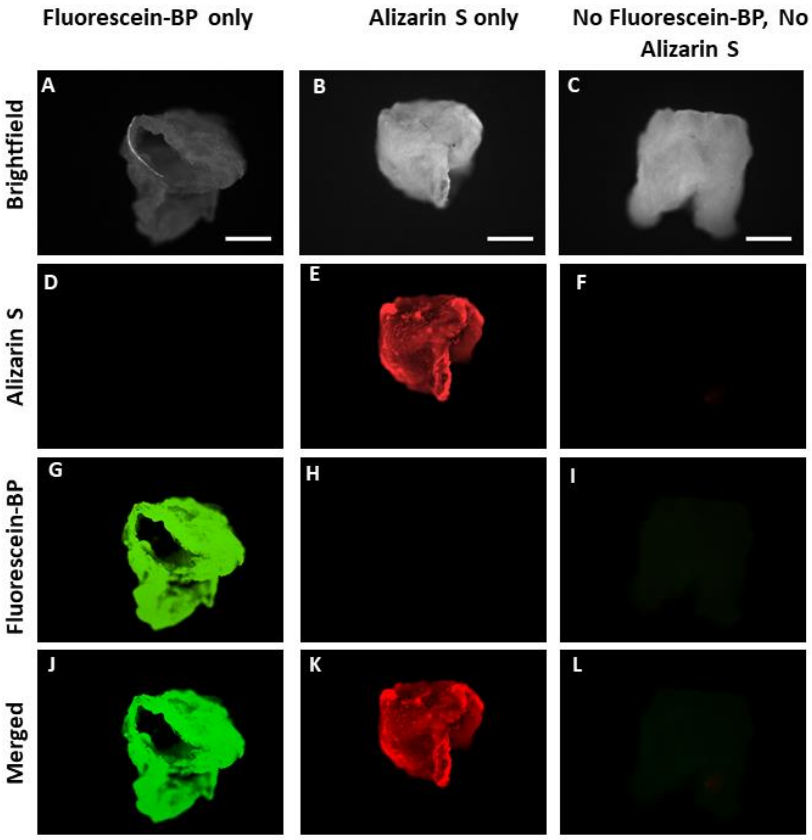

SI Figure 6: Ex vivo aorta ring controls. Rat aortic rings were incubated for 2 days in fresh control media followed by calcification media (3.0 mM Pi) for 7 days. The rings were subsequently incubated with either Fluorescein-BP (1 µM, 2 hours) and then fixed with NBF (10%, 15 minutes) or fixed with NBF (10%, 15 minutes), permeabilised in KOH (1%, 1 hour) and then incubated with Alizarin S (0.00005% Alizarin S in 1% KOH, 24 hours). Brightfield (**A**, **B**, **C**), Fluorescence (**G**, **H**, **I**), Alizarin S (**D**, **D**, **F**). Merged images (**J**, **K**, **L**) recorded using both Alizarin S and Fluorescein-BP channels. Data shown are representative of at least 3 independent experiments yielding comparable results. Scale bars = 250 µm.

Human tissue samples

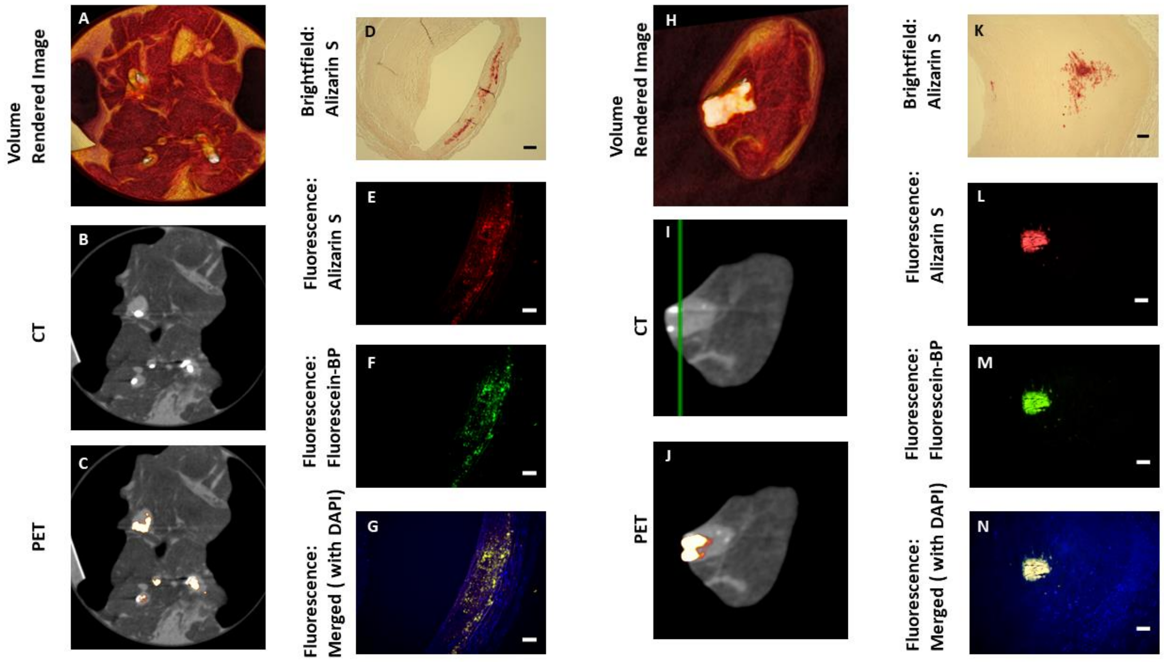

SI Figure 7: Determination of calcification in human vascular tissue (calcified and control). (**A**, **H**) Volume rendered image; (**B**, **I**) CT image; (**C**, **J**) PET image, with $Na^{18}F$ tracer, of vascular tissue. (**D**, **E**, **F**, **G**, **K**, **L**, **M**, **N**) Brightfield and Fluorescence (Alizarin S, Fluorescein-BP, DAPI) images of calcified tissues. Sections are incubated with Fluorescein-BP (1 µM, 2 hours), Alizarin S (2%, 10 minutes) and DAPI (500 nM, 5 minutes) and subsequently imaged. Scale bars = 2.0 mm for Brightfield and 100 µm for Fluorescence.

References

1. Gavin, C. T. *et al.* Novel Methods of Determining Urinary Calculi Composition: Petrographic Thin Sectioning of Calculi and Nanoscale Flow Cytometry Urinalysis. *Sci. Rep.* **6,** 19328 (2016).
2. Zhu, D., Mackenzie, N. C. W., Millán, J. L., Farquharson, C. & MacRae, V. E. The Appearance and Modulation of Osteocyte Marker Expression during Calcification of Vascular Smooth Muscle Cells. *PLoS ONE* **6**, e19595 (2011).
3. Lee, Y.-K., Song, J., Lee, S.-B., Kim, K.-M., Choi, S.-H., Kim, C.-K., LeGeros, R. Z. & Kim, K.-N. Proliferation, differentiation, and calcification of preosteoblast-like MC3T3-E1 cells cultured onto noncrystalline calcium phosphate glass. *J. Biomed. Mater. Res.* **69A**, 188–195 (2004).
